# Supplementary material for: Loss of H3K9 trimethylation alters chromosome compaction and transcription factor retention during mitosis
Source: Nat Struct Mol Biol. 2023 Mar 20;30(4):489–501. doi: 10.1038/s41594-023-00943-7 (PMC10113154; doi:10.1038/s41594-023-00943-7)
Supplement: Supplementary file 1 — Supplementary figs. 1–6, methods, tables 1–3, references and source data for Supplementary Fig. 4c. [file 41594_2023_943_MOESM1_ESM.pdf]

nature structural & molecular biology

Article https://doi.org/10.1038/s41594-023-00943-7

Loss of H3K9 trimethylation alters chromosome compaction and transcription factor retention during mitosis

In the format provided by the authors and unedited

Supplemental Information

Loss of H3K9 tri-methylation alters chromosome compaction and transcription factor retention during mitosis

Djeghloul, D., *et al.*

Supplemental Figure S1
Supplemental Figure S2
Supplemental Figure S3
Supplemental Figure S4
Supplemental Figure S5
Supplemental Figure S6
Supplemental Methods
Supplemental Table 1
Supplemental Table 2
Supplemental Table 3
Supplemental References

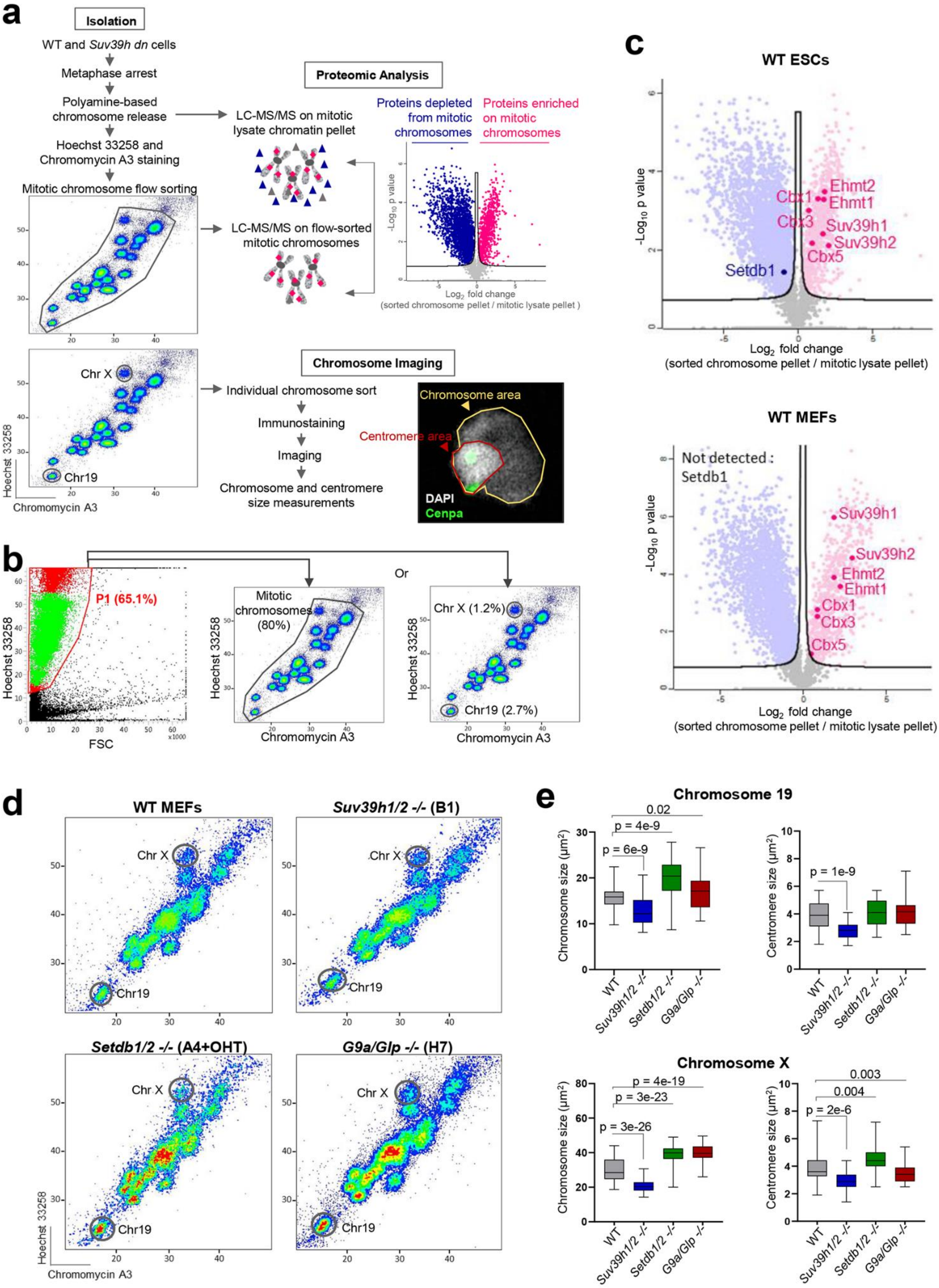

Figure S1: (a) Scheme of experimental strategy used to isolate native metaphase chromosomes from WT and *Suv39h dn* cells, and identify proteins bound to mitotic chromatin in each cell line. Hoechst 33258 and Chromomycin A3

bivariate karyotype was assessed by flow cytometry and the gates used to sort all chromosomes, chromosome 19, or the X chromosome are indicated. Proteomic analysis was performed using LC-MS/MS on total mitotic cell lysate pellet, or on flow-purified chromosomes, to identify proteins enriched on metaphase chromosomes. Chromosome 19 and X size measurements were performed using Fiji/imageJ software to estimate chromosome (total DAPI) and centromere (DAPI high) areas, as indicated. (b) Gating strategy for sorting mitotic chromosomes, percentage of each gate is indicated (c) Volcano plots of proteins detected as being significantly enriched (red), depleted (blue) or not significantly enriched (grey) on sorted chromosomes relative to mitotic lysate pellet for WT ESCs (upper plot) or WT MEFs (lower plot). H3K9 KMTs and HP1 proteins are highlighted on the volcano plots. Statistical analysis was performed using unpaired two tailed Student's t-test, permutation-based FDR < 0.05, n = 3 independent experiments each measured in duplicate, see Methods for details). Proteins were plotted as Log2 fold change (LFQ intensity of sorted chromosome pellet /LFQ intensity of mitotic lysate pellet) and significance (-Log10 p) using Perseus software. (d) Flow karyotypes of mitotic chromosomes isolated from WT, *Suv39h1/2 -/-* (B1), *Setdb1/2 -/-* (A4+OHT), or *G9a/Glp -/-* (H7) MEFs. Gates used to isolate chromosomes 19 and X from each cell line are indicated. (e) Chromosome 19 (upper panel) and X (lower panel) size measurements from WT, *Suv39h1/2 -/-* (B1), *Setdb1/2 -/-* (A4+OHT), or *G9a/Glp -/-* (H7) MEFs. Box plots show area measurements of individual chromosomes and centromeres for each MEF line. Minimum, lower quartile, median, upper quartile and maximum values are indicated. n = minimum 100 chromosomes analysed for each condition over three independent experiments. P-values of statistically significant changes compared to WT, measured by unpaired two tailed Student's t-tests, are indicated. Source data, including the precise number of chromosomes analysed, are provided in Supplemental Data 3.

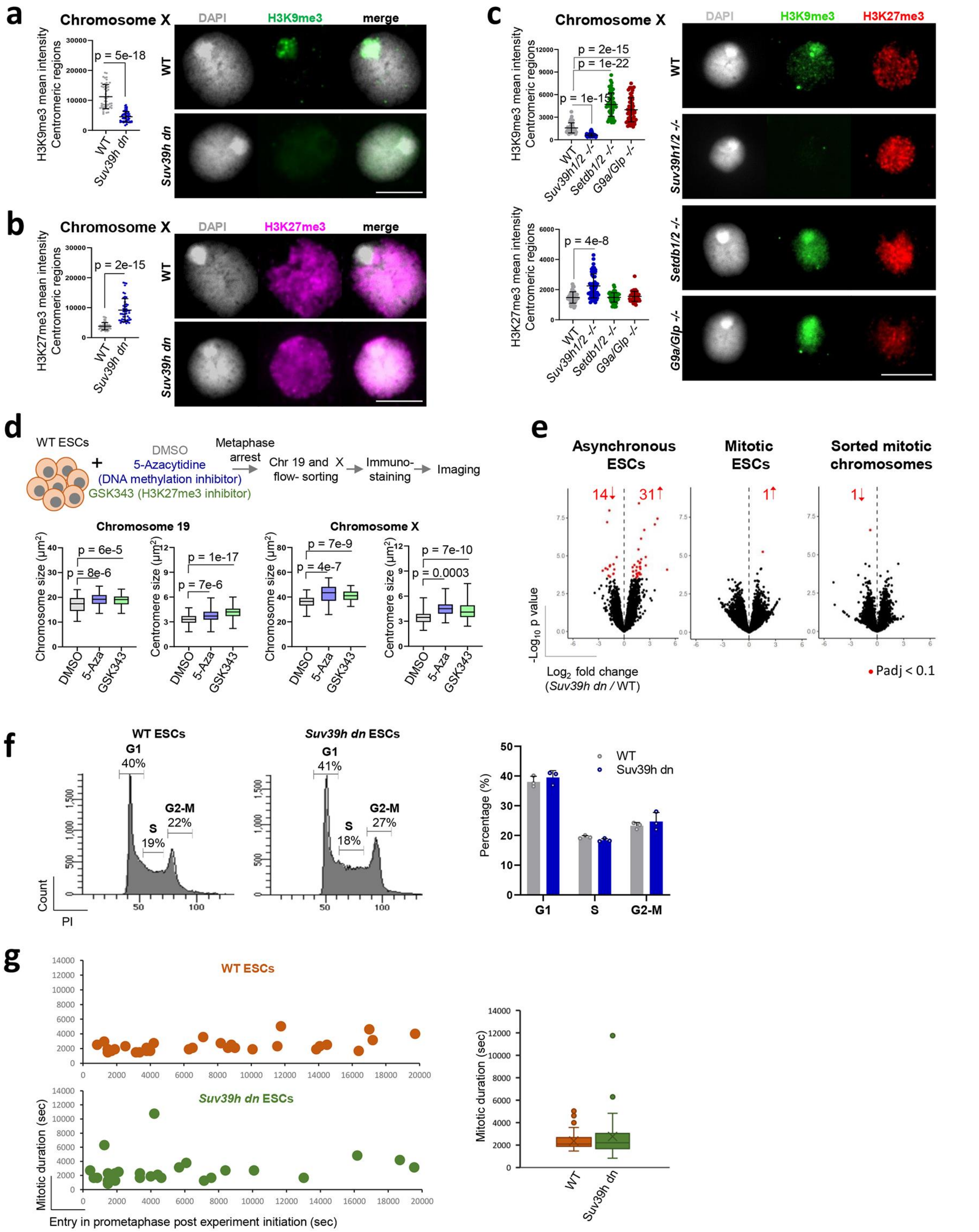

Figure S2: (a,b) Representative images (right panel) of immunofluorescence labelling of histone H3K9me3 (a) (green) or histone H3K27me3 (b) (pink) on mouse chromosome X isolated from WT or *Suv39h dn* ESCs where DAPI counterstain is shown in light grey. Scale bars = 5 µm. Plots (left of the images) show H3K9me3 (a) or H3K27me3 (b) mean intensities measured at centromeric regions. Mean ± SD are shown, n = minimum 50 chromosomes over three independent

experiments. P-values of statistically significant changes, measured by unpaired two tailed Student's t-tests, are indicated. (c) Representative images (right panel) of histone H3K9me3 (green) and H3K27me3 (red) co-immunolabelling on mouse chromosome X isolated from WT, *Suv39h1/2 -/-* (B1), *Setdb1/2 -/-* (A4+OHT), or *G9a/Glp -/-* (H7) MEFs, where DAPI counterstain is shown in light grey. Scale bars = 5 µm. H3K9me3 mean intensities (upper plot) was measured at centromeric regions for each MEF line, mean ± SD are shown, n = minimum 50 chromosomes over three independent experiments. P-values of statistically significant changes, measured by unpaired two tailed Student's t-tests, are indicated. H3K27me3 mean intensities (lower plot) was measured at centromeric regions for each MEF line, mean ± SD are shown, n = minimum 50 chromosomes over three independent experiments. P-values of statistically significant changes, measured by unpaired two tailed Student's t-tests, are indicated. (d) Experimental strategy (top panel) used to measure mitotic chromosome size of WT ESCs after treatment with DNA methylation or PRC2 inhibitors (5-Aza or GSK343 respectively). Chromosome and centromere sizes were calculated for each condition. Box plots show area measurements of individual chromosomes and centromeres for each condition. Minimum, lower quartile, median, upper quartile and maximum values are indicated. n = 100 chromosomes over three independent experiments. (a-d) P-values of statistically significant changes, measured by unpaired two tailed Student's t-tests, are indicated. Source data, including the precise number of chromosomes analysed, are provided in Supplemental Data 3. (e) Volcano plots showing differential accessibility analysis of ATAC-seq peaks for *Suv39h dn* vs WT ESCs. Differential accessibility analysis was performed with voom-limma (Padj < 0.1). (f) Representative cell cycle profiles of WT and *Suv39h dn* ESCs determined by staining with propidium iodide (PI). Values indicate percentage of cells in G1, S, and G2/M phases. Bar graph shows the mean percentage in each phase of the cell cycle measured for each cell type, n = 3 independent experiments, error bars show SD. (g) Mitotic duration analysis for WT (upper plot) and *Suv39h dn* (lower plot) ESCs using live cell imaging. Mitotic duration was calculated as the time difference between the entry into prometaphase and late anaphase for each cell. Mitotic duration was calculated for cells beginning division at different time points after initiating imaging to ensure duration was consistent throughout the experiment. Box plot (right panel) summarises the measurements of mitotic duration for WT and *Suv39h dn* ESCs. Minimum, lower quartile, median, upper quartile and maximum values are indicated. n = 30 cells for each condition. (f,g) No statistically significant changes were observed using unpaired two tailed Student's t-tests. (f, g) Source data are provided in Supplemental Data 3.

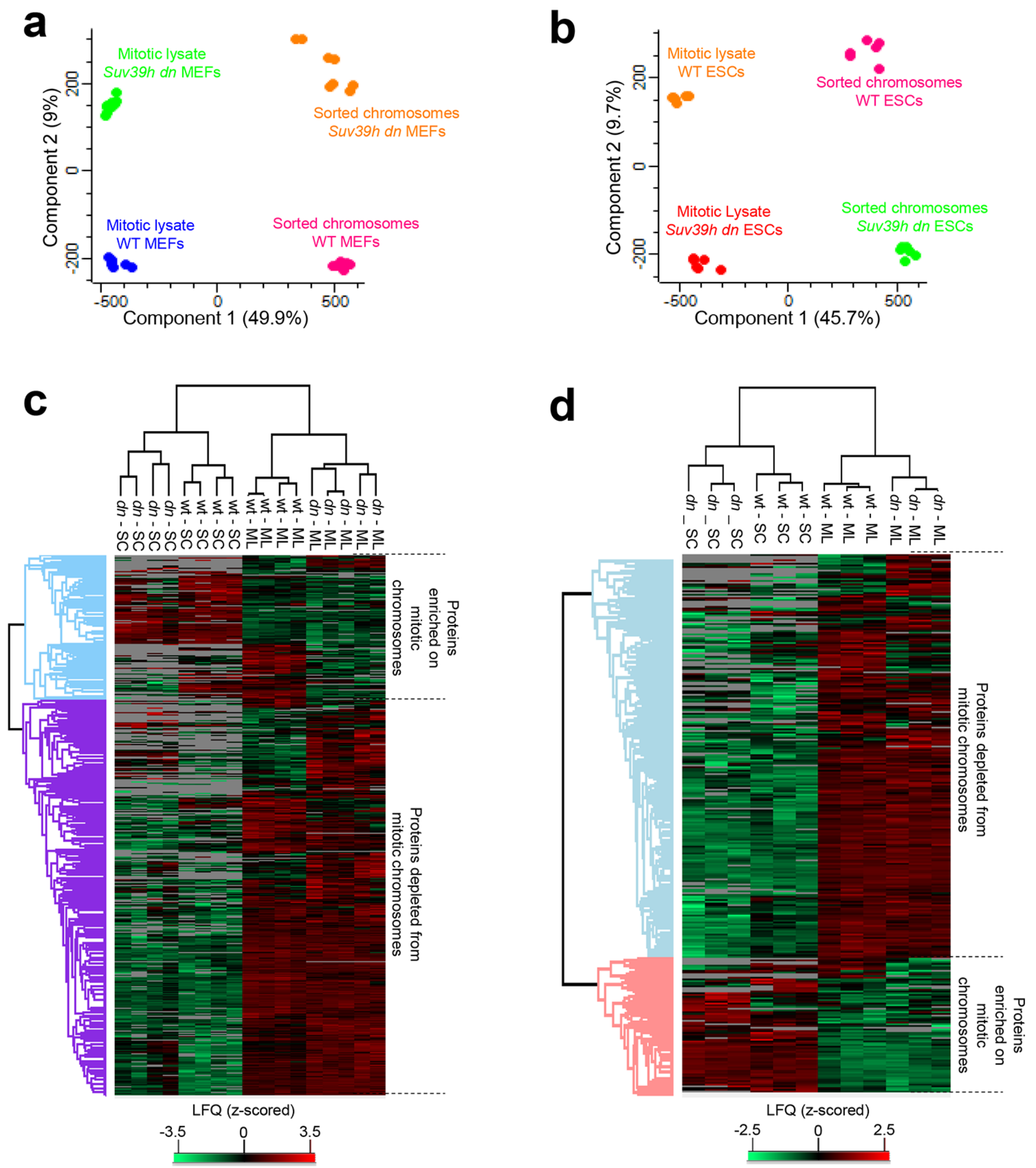

Figure S3: (a,b) Principal component analysis (PCA) of proteomic datasets. (c,d) Heatmap and hierarchical clustering analysis (HCA) of significantly changed protein hits (two-sided student's t-test, FDR 0.05) for MEF (c) and ESC (d) samples. Colour scale provided displays z-scored label-free quantification (LFQ) intensities; grey represents missing values (ie not detected in that sample), *dn* = *Suv39h dn*, SC = Sorted chromosomes, ML = Mitotic lysate pellet.

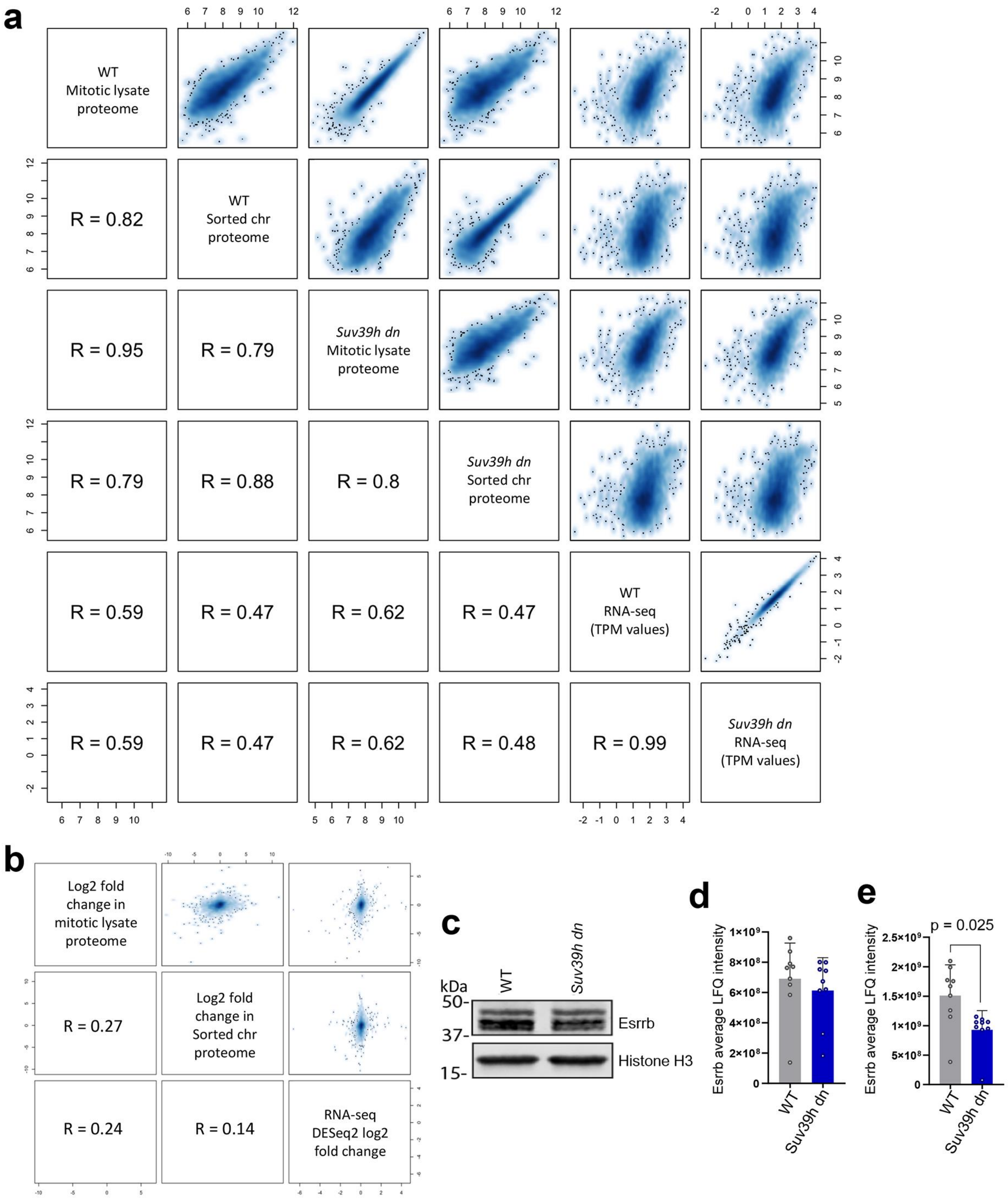
a
WT
Mitotic lysate
proteome
R = 0.82
WT
Sorted chr
proteome
R = 0.95
R = 0.79
Suv39h dn
Mitotic lysate
proteome
R = 0.79
R = 0.88
R = 0.8
Suv39h dn
Sorted chr
proteome
R = 0.59
R = 0.47
R = 0.62
R = 0.47
WT
RNA-seq
(TPM values)
R = 0.59
R = 0.47
R = 0.62
R = 0.48
R = 0.99
Suv39h dn
RNA-seq
(TPM values)
b
Log2 fold
change in
mitotic lysate
proteome
R = 0.27
Log2 fold
change in
Sorted chr
proteome
R = 0.24
R = 0.14
RNA-seq
DESeq2 log2
fold change
c
kDa
WT
Suv39h dn
50-
37-
15-
Esrrb
Histone H3
d
Esrrb average LFQ intensity
WT
Suv39h dn
e
p = 0.025
Esrrb average LFQ intensity
WT
Suv39h dn

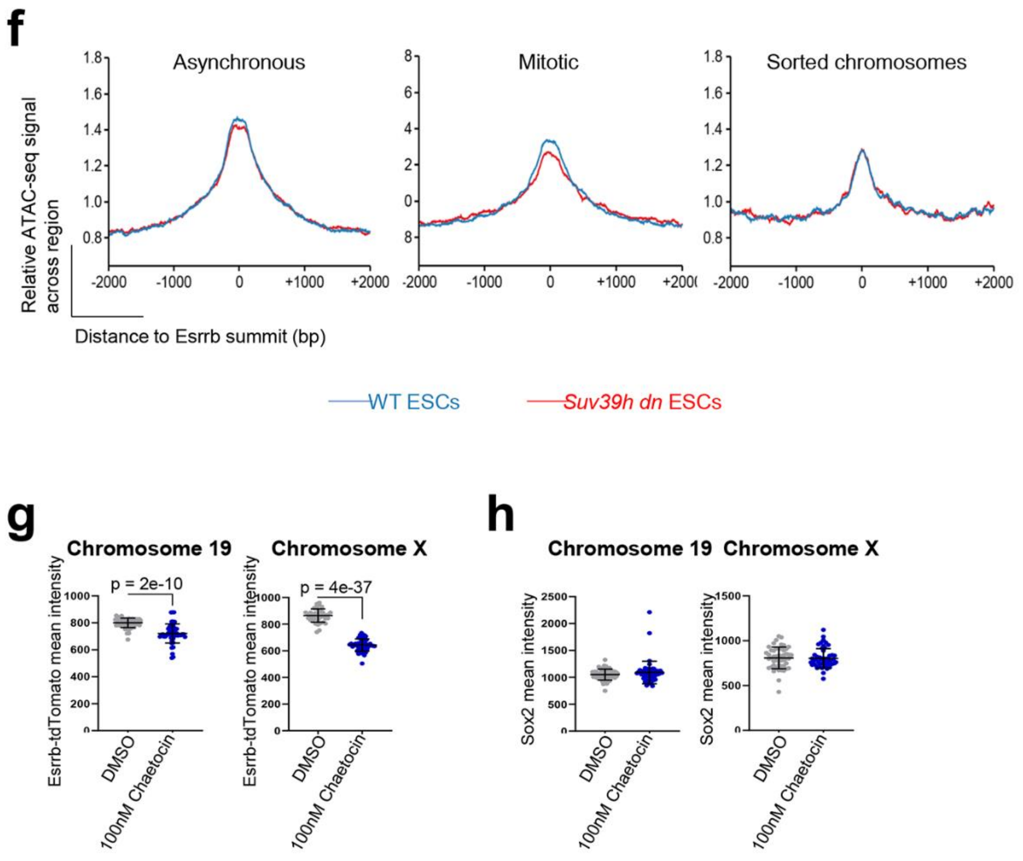

Figure S4: (a) Pairwise correlations (R values from Spearman correlation) between proteomics LFQ values (this study) and polyA RNAseq TPM (Transcripts per million) values in ESCs (dataset from[1]). In total, 5664 genes were mapped between proteomics and transcriptomics experiments based on gene symbols. (b) Pairwise correlations (R values from Spearman correlation) between log2 fold changes in proteomics LFQ values (this study) and DESeq2 log2 fold changes in polyA RNAseq values (dataset from[1]) comparing *Suv39h dn* vs WT ESCs. (c) Western blot of Esrrb in WT and *Suv39h dn* asynchronous ESCs, representative of three biological replicates. Histone H3 was used as a loading control for the western blot. The uncropped image is provided at the end of this Supplementary information file. (d,e) Average LFQ intensities of Esrrb in WT and *Suv39h dn* mitotic lysates (d) and sorted chromosomes (e). Mean + SD is shown, n = 3 independent experiments each measured in duplicate. (d,e) P-value of statistically significant change, measured by unpaired two tailed Student's t-tests, is indicated. Source data are provided in Supplemental Data 3. (f) Trend of ATAC-seq accessibility around Esrrb bookmarked binding sites in WT and *Suv39h dn* asynchronous ESCs (left), mitotic ESCs (middle) and sorted chromosomes (right). Esrrb peak locations and bookmarking status are taken from[2]. (g,h) Esrrb (g) and Sox2 (h) mean intensities on sorted chromosomes 19 and X from Esrrb-tdTomato ES cell line following treatment with DMSO or 100 nM Chaetocin. Plots show mean ± SD, n = minimum 50 chromosomes over three independent experiments. P-values of statistically significant changes, measured by unpaired two tailed Student's t-tests, are indicated. Source data, including the precise number of chromosomes analysed, are provided in Supplemental Data 3.

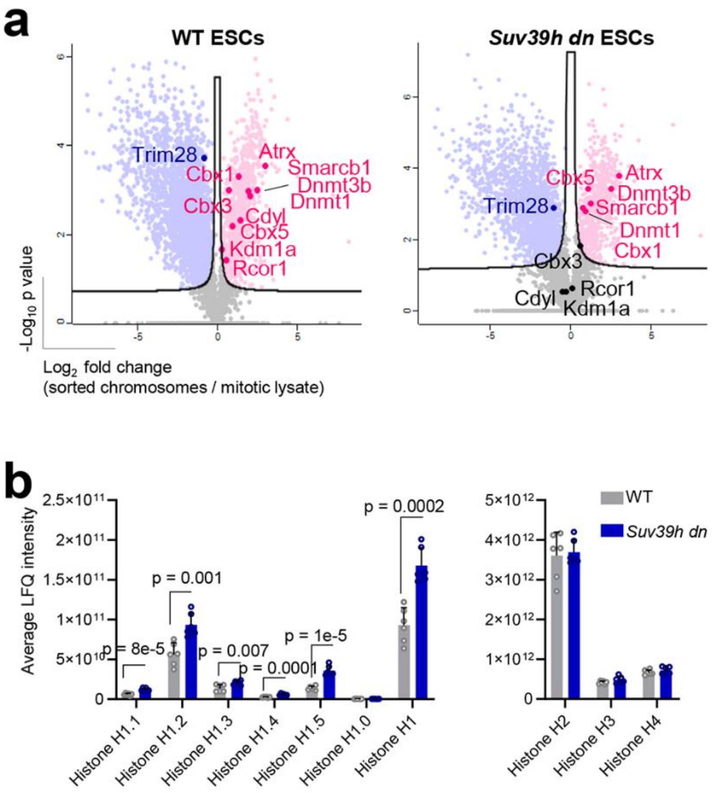

Figure S5: (a) Volcano plots as in Figure 3d, highlighting H3K9me3-associated factors that are enriched (red), depleted (blue) or not significantly enriched (black) on WT (left) or *Suv39h dn* (right) ESC mitotic chromosomes versus mitotic lysates. (b) Average LFQ intensity of different histone H1 variants and total histone H1, H2, H3, and H4 in the sorted chromosome samples of WT (grey) and *Suv39h dn* (blue). Mean + SD is shown, n = 3 independent experiments each measured in duplicate. P-values of statistically significant changes, measured by unpaired two tailed Student's t-tests, are indicated. Source data are provided in Supplemental Data 3.

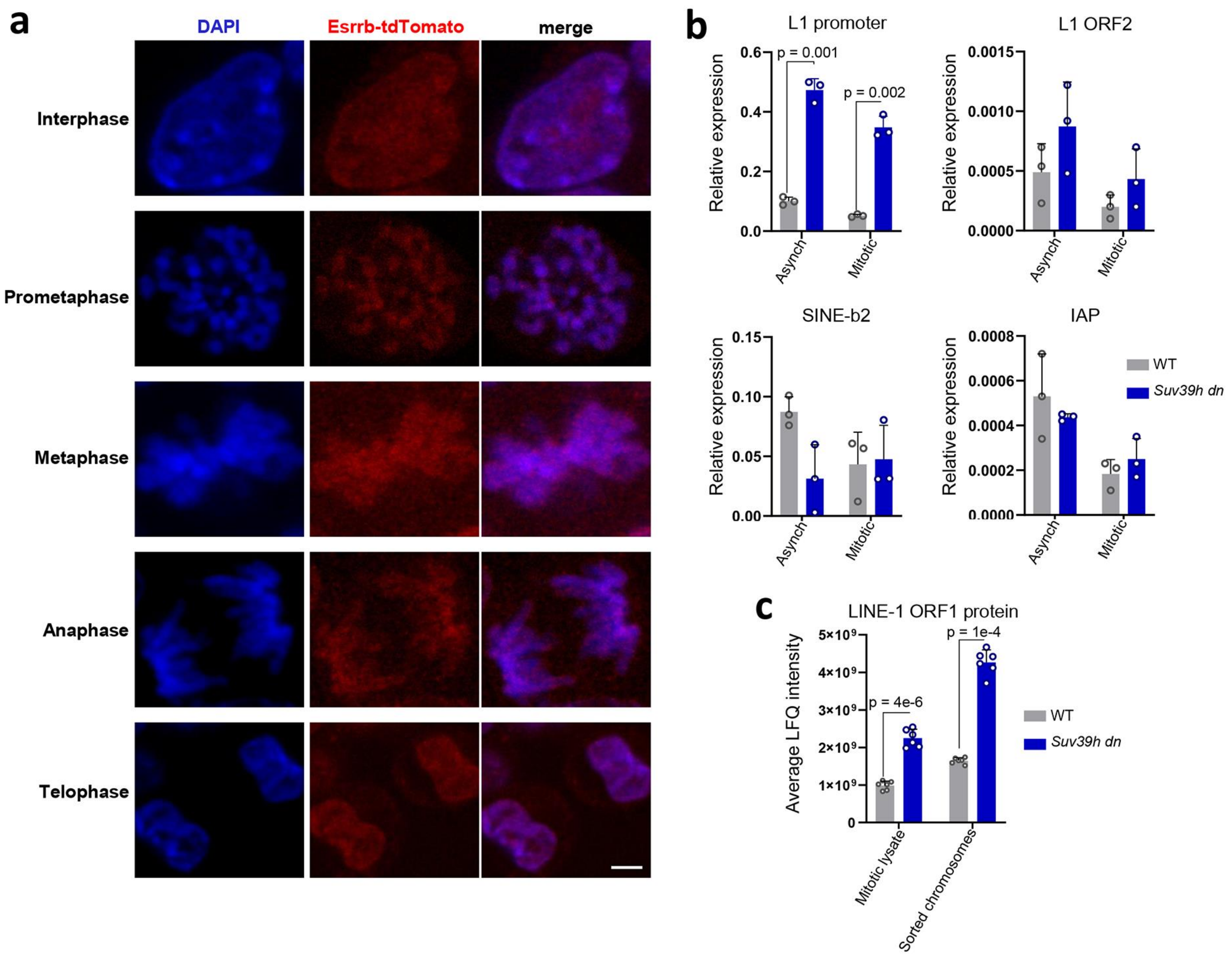

Figure S6: (a) Representative images of Esrrb-tdTomato ESCs (DSG+PFA double fixed) in different phases of the cell cycle. DAPI counterstain in blue. Scale bars = 5 µm, representative of three biological replicates. (b) qRT-PCR expression analysis of LINE-1, SINE-b2 and IAP repeat elements in WT and *Suv39h dn* asynchronous and mitotic ESCs. Mean + SD is shown, n = 3 independent experiments, P-values of statistically significant changes, measured by unpaired two tailed Student's t-tests, are indicated. (c) Average LFQ intensity of L1ORF1 in mitotic lysates and sorted chromosome samples of WT and *Suv39h dn* ESCs. Mean + SD is shown, n = 3 independent experiments each measured in duplicate. P-values of statistically significant changes, measured by unpaired two tailed Student's t-tests, are indicated. (b,c) Source data are provided in Supplemental Data 3.

Supplemental methods

Cell cycle analysis using PI staining

WT and *Suv39h dn* ESCs (10^6 cells) were fixed with ice-cold 70% ethanol, washed twice with PBS, resuspended in staining buffer (0.05 mg/ml PI, 1 mg/ml RNaseA, 0.05% NP40), and incubated for 10 min at room temperature (RT) and 20 min on ice. PI signal was analysed in linear mode with a BD Fortesa flow cytometer and BD DIVA software.

Mitotic duration analysis using live cell imaging

WT and *Suv39h dn* ESCs were plated in ESC medium in gelatin-coated 8 Well Ibidi µ-Slides (Ibidi, 80826) 24 h before imaging. Next day, cells were switched to phenol red-free medium (31053-028, Gibco) containing 15% FBS, 1X non-essential amino acids and sodium pyruvate, 2 mM L-glutamine, 100 µM 2-mercaptoethanol, antibiotics, and LIF, buffered with 20 mM HEPES, and incubated with 1 µM SiR-DNA (SC007, SpiroChrome) for 30 min before imaging. Time-lapse images were acquired on an Olympus IX83 microscope equipped with a Yokogawa CSU-W1 spinning disk and Hamamatsu ORCA-Flash 4.0 camera using cellSens Dimension software (version 2.3). Z-stacks were collected every 210 s with a step-size of 0.5 µm using a UPlanSApo 60x/1.35 Oil objective lens, with an environmental chamber kept at 37°C with a 5% CO2 supply. Mitotic duration analysis was performed in Fiji[3] using a script that facilitated user selection of time-points corresponding to the entry in prometaphase and late anaphase, followed by calculation of the duration between the stages.

Quantitative RT-PCR

Total RNA was extracted from asynchronous or metaphase-arrested WT and *Suv39h dn* ESCs ($5x10^5$ cells) using RNeasy mini Kit (74106, Qiagen). Extracted RNA was treated twice with Turbo DNA free (AM1907, Invitrogen) according to the manufacturer's instructions and reverse transcribed using SuperScript III (18080085, Invitrogen) and random primers (Invitrogen). Real time PCR was performed in technical triplicate for each sample, using SYBR Green PCR Master Mix (204145, Qiagen) on a real time PCR machine (Bio-Rad CFX96 system with CFX manager software). Values were normalized to *β-actin* and *Gapdh*. Primer sequences used for gene expression analysis are listed in Supplemental Table 3.

Western blot

WT and *Suv39h dn* asynchronous ESCs (10^6 cells) were pelleted, resuspended in 100 µl cold RIPA buffer (50 mM Tris-HCl pH 8.8, 150 mM NaCl, 1% Triton X-100, 0.5% sodium deoxycholate, 0.1% SDS, 1 mM EDTA, 3 mM $MgCl_2$) containing 1× protease inhibitor cocktail (11873580001, Roche) and 1.25 U/µl Benzonase

(E1014, Sigma) and incubated for 20 min at RT. Samples were mixed with 100 µl of 2× Laemmli sample buffer (65.8 mM Tris-HCl pH 6.8, 2.2% SDS, 22.2% glycerol, 0.01% bromophenol blue and 710 mM 2-mercaptoethanol) and denatured (10 min at 95 °C). Standard protocols were followed for western blots. Immobilon Block-FL (WBAVDFL01, Millipore) was used as a fluorescent blocker. The following antibodies and dilutions were used: anti-Esrrb (PP-H6705-00, Perseus Proteomics, 1:1000) with anti-mouse-Alexa790 (A11371, Invitrogen, 1:10000) and anti-Histone H3 (61476, Active Motif, 1:5000) with anti-rabbit-Alexa680 (A21109, Invitrogen, 1:10000). Detection was carried out using the LI-COR detection system with Image Studio software.

ATAC-seq library	Primer name	Primer sequence	Index sequence
All	Ad1	AATGATACGGCGACCACCGAGATCTACACTCGTCGGCAGCGTCAGATGTG	-
WT_Asynchr_1	Ad2.1	CAAGCAGAAGACGGCATACGAGATTCGCCTTAGTCTCGTGGGCTCGGAGATGT	TAAGGCGA
WT_Asynchr_2	Ad2.3	CAAGCAGAAGACGGCATACGAGATTTCTGCCTGTCTCGTGGGCTCGGAGATGT	AGGCAGAA
Suv39h dn_Asynchr_1	Ad2.2	CAAGCAGAAGACGGCATACGAGATCTAGTACGGTCTCGTGGGCTCGGAGATGT	CGTACTAG
Suv39h dn_Asynchr_2	Ad2.5	CAAGCAGAAGACGGCATACGAGATAGGAGTCCGTCTCGTGGGCTCGGAGATGT	GGACTCCT
WT_Mitotic_1	Ad2.4	CAAGCAGAAGACGGCATACGAGATGCTCAGGAGTCTCGTGGGCTCGGAGATGT	TCCTGAGC
WT_Mitotic_2	Ad2.6	CAAGCAGAAGACGGCATACGAGATCATGCCTAGTCTCGTGGGCTCGGAGATGT	TAGGCATG
Suv39h dn_Mitotic_1	Ad2.7	CAAGCAGAAGACGGCATACGAGATGTAGAGAGGTCTCGTGGGCTCGGAGATGT	CTCTCTAC
Suv39h dn_Mitotic_2	Ad2.14	CAAGCAGAAGACGGCATACGAGATACAGTGGTGTCTCGTGGGCTCGGAGATGT	ACCACTGT
WT_SC_1	Ad2.8	CAAGCAGAAGACGGCATACGAGATCCTCTCTGGTCTCGTGGGCTCGGAGATGT	CAGAGAGG
WT_SC_2	Ad2.19	CAAGCAGAAGACGGCATACGAGATCCCAACCTGTCTCGTGGGCTCGGAGATGT	AGGTTGGG
Suv39h dn_SC_1	Ad2.9	CAAGCAGAAGACGGCATACGAGATAGCGTAGCGTCTCGTGGGCTCGGAGATGT	GCTACGCT
Suv39h dn_SC_2	Ad2.20	CAAGCAGAAGACGGCATACGAGATCACCACACGTCTCGTGGGCTCGGAGATGT	GTGTGGTG

Supplemental Table 1: Primers for ATAC-seq library amplification and indexing. Sequences were obtained from[4] and ordered from Sigma-Aldrich (HPLC purified). SC, sorted chromosomes.

Locus	F primer	R primer	Esrrb bookmarking state (Festuccia et al., 2016)	Reference
Capn2	GCTCTGTCTCACGATTCTGG	CTTCCATGATCCGGTCCCTT	bookmarked (Asynchronous+Mitosis)	designed in this study
Esrrb	CGGCTGGTATCACCTGATTT	GCTTTGTCCTCTTTGCCAAT	bookmarked (Asynchronous+Mitosis)	Festuccia et al., (2016)[5]
Jam2_s1	CTGGGCCTGGACCTAAACTC	TCCCACCCTGCTTAGTGTTTT	bookmarked (Asynchronous+Mitosis)	Festuccia et al., (2016)[5]
Jam2_s2	ACCACGTCATTCTGTCCCTT	CCTTGCGAACACTCCATCAC	bookmarked (Asynchronous+Mitosis)	designed in this study
Tbx3	GGTGGACACTTTTGGGAATG	GGTTTCTCCAGTTGACCATGA	bookmarked (Asynchronous+Mitosis)	designed in this study
Rex1	AGGACGGATATGGCTTTGCG	CCTCGACCTTCCTCTGTCCT	bookmarked (Asynchronous+Mitosis)	Festuccia et al., (2016)[5]
Tet2	ACTTTGAATTGGGCAGGTCTCA	ATCTCCTGTCCTGGCTCTGA	bookmarked (Asynchronous+Mitosis)	Festuccia et al., (2016)[5]
Mgat3	CCTGTGGTGACACTGTGGAA	CACCCAGGTCATACCCTTGA	Lost (Asynchronous only)	Festuccia et al., (2016)[5]
Twistnb	CCAGTTGTTTCCGGGAGAAT	GCTGCCTGAGACTCCATGAC	Lost (Asynchronous only)	Festuccia et al., (2016)[5]
Esrrb 3'	ACTCCTCCCCTTACCCCTGT	GGCTGTGGTCACTGCATCTA	No Esrrb (negative control)	Festuccia et al., (2016)[5]
Actb (ChIP)	CCGTTCCGAAAGTTGCCTT	CGCCGCCGGGTTTTATA	No Esrrb, No K9 (negative control)	Festuccia et al., (2016)[5]

Supplemental Table 2: Primers for Esrrb ChIP-qPCR.

	F primer	R primer	Reference
L1 promoter	ACTGCGGTACATAGGGAAGC	TGTGATCCACTCACCAGAGG	Bulut-Karslioglu et al., (2014)[1]
L1 ORF2	ACCTGGACGAAATGGACAAA	CATCTGGTCCTGGGCTTTT	Bulut-Karslioglu et al., (2014)[1]
IAP LTR	AGGGTGGTTCTCTACTCCAT	GAACACCACAGACCAGAATC	Ryu et al., (2011)[6]
SINE B2	GGCTGGTGAGATGGCTCAGT	TACACTGTAGCTGTCTTCAGACA	Allen et al., (2004)[7]
β-Actin	CATCCGTAAAGACCTCTATGCCAAC	ATGGAGCCACCGATCCACA	This study
Gapdh	GCGAGACCCCACTAACATCA	CACACCCATCACAAACATGG	This study

Supplemental Table 3: Primers used for quantitative RT-PCR.

Supplemental References

1. Bulut-Karslioglu, A. *et al.* Suv39h-dependent H3K9me3 marks intact retrotransposons and silences LINE elements in mouse embryonic stem cells. *Mol Cell* **55**, 277-290 (2014).
2. Festuccia, N. *et al.* Transcription factor activity and nucleosome organization in mitosis. *Genome Res* **29**, 250-260 (2019).
3. Schindelin, J. *et al.* Fiji: an open-source platform for biological-image analysis. *Nat Methods* **9**, 676-682 (2012).
4. Buenrostro, J.D., Giresi, P.G., Zaba, L.C., Chang, H.Y. & Greenleaf, W.J. Transposition of native chromatin for fast and sensitive epigenomic profiling of open chromatin, DNA-binding proteins and nucleosome position. *Nat Methods* **10**, 1213-1218 (2013).
5. Festuccia, N. *et al.* Mitotic binding of Esrrb marks key regulatory regions of the pluripotency network. *Nat Cell Biol* **18**, 1139-1148 (2016).
6. Ryu, S.H., Kang, K., Yoo, T., Joe, C.O. & Chung, J.H. Transcriptional repression of repeat-derived transcripts correlates with histone hypoacetylation at repetitive DNA elements in aged mice brain. *Exp Gerontol* **46**, 811-818 (2011).
7. Allen, T.A., Von Kaenel, S., Goodrich, J.A. & Kugel, J.F. The SINE-encoded mouse B2 RNA represses mRNA transcription in response to heat shock. *Nat Struct Mol Biol* **11**, 816-821 (2004).

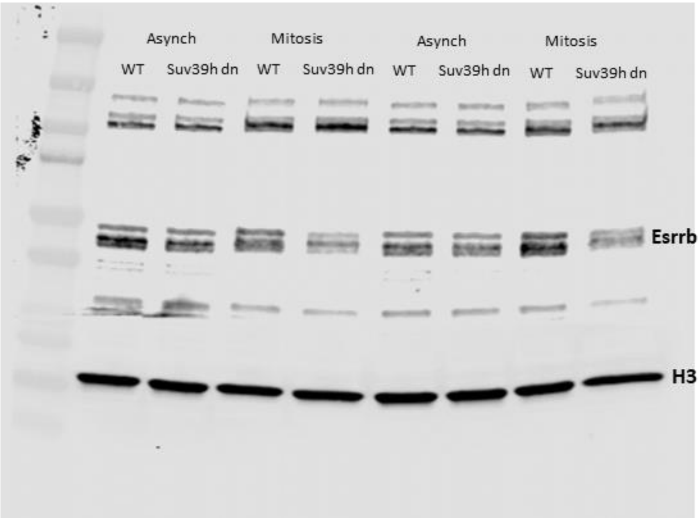

Uncropped WB - Source data for Figure S4c.
