# Supplementary material for: Loss of H3K9 trimethylation alters chromosome compaction and transcription factor retention during mitosis
Source: Nat Struct Mol Biol. 2023 Mar 20;30(4):489–501. doi: 10.1038/s41594-023-00943-7 (PMC10113154; doi:10.1038/s41594-023-00943-7)
Supplement: Supplementary file 2 — Reporting summary. [file 41594_2023_943_MOESM2_ESM.pdf]

nature portfolio

Corresponding author(s): Amanda Fisher and Dounia Djeghloul

Last updated by author(s): Jan 19, 2023

Reporting Summary

Nature Portfolio wishes to improve the reproducibility of the work that we publish. This form provides structure for consistency and transparency in reporting. For further information on Nature Portfolio policies, see our Editorial Policies and the Editorial Policy Checklist.

Statistics

For all statistical analyses, confirm that the following items are present in the figure legend, table legend, main text, or Methods section.

n/a	Confirmed	
☐	☒	The exact sample size (*n*) for each experimental group/condition, given as a discrete number and unit of measurement
☐	☒	A statement on whether measurements were taken from distinct samples or whether the same sample was measured repeatedly
☐	☒	The statistical test(s) used AND whether they are one- or two-sided<br>*Only common tests should be described solely by name; describe more complex techniques in the Methods section.*
☐	☒	A description of all covariates tested
☒	☐	A description of any assumptions or corrections, such as tests of normality and adjustment for multiple comparisons
☐	☒	A full description of the statistical parameters including central tendency (e.g. means) or other basic estimates (e.g. regression coefficient) AND variation (e.g. standard deviation) or associated estimates of uncertainty (e.g. confidence intervals)
☐	☒	For null hypothesis testing, the test statistic (e.g. *F*, *t*, *r*) with confidence intervals, effect sizes, degrees of freedom and *P* value noted<br>*Give P values as exact values whenever suitable.*
☒	☐	For Bayesian analysis, information on the choice of priors and Markov chain Monte Carlo settings
☒	☐	For hierarchical and complex designs, identification of the appropriate level for tests and full reporting of outcomes
☒	☐	Estimates of effect sizes (e.g. Cohen's *d*, Pearson's *r*), indicating how they were calculated

Our web collection on statistics for biologists contains articles on many of the points above.

Software and code

Policy information about availability of computer code

Data collection

BD FACS software (v1.2.0.142) and BD DIVA (v8.0.1) were use to collect FACS data.
Micro-Manager (v1.4.22, IX70 microscope), LAS-AF (2.7.3.9723, SP5 II microscope), cellSens Dimension (v2.3, IX83 microscope) were used to collect imaging data.
Image Studio software (v4.0.21) was used for fluorescent western blot imaging.
Quantitative real-time PCR data was collected using Bio-Rad CFX Manager software (v3.1)
Sequencing data were collected on an Illumina NextSeq500.

Data analysis

ImageJ/Fiji (version 1.52p) was used for image analysis including chromosome and centromere size measurements and mitotic duration analysis.
Image deconvolution was performed with Huygens Professional (v19.10, Scientific Volume Imaging, http://svi.nl), using the CMLE algorithm.
Proteomics data were analyzed using the Label-Free Quantification algorithm in the MaxQuant software platform (v1.6.2.3). The Perseus software (v1.6.2.2 and v1.6.7.0) was used for both statistical analysis and data visualization of the proteomics results.
ATAC-seq data were processed using RTA (v2.11.3), reads were demultiplexed with bcl2fastq2 (v2.20.0), trimmed with Trim Galore! (trim_galore_v0.4.4; --trim-n --paired; www.bioinformatics.babraham.ac.uk/projects/trim_galore) and aligned with bowtie2 (bowtie2/2.2.9; -p8 -t -very-sensitive -X 2000). Bam files were processed with Samtools (v1.2). Duplicates were marked with Picardtools MarkDuplicates (v1.90). The bamQC function from R/Bioconductor package ATACseqQC (v1.10.4) was used to keep properly mapped paired-end reads, and remove duplicates and mitochondrial alignments. Chromosome-wide accessibility profiles and accessibility trend plots were generated from the resulting bam files in Seqmonk (v1.48.0, www.bioinformatics.babraham.ac.uk/projects/seqmonk). Nucleosome-free fragments were extracted using the alignmentSieve function from deepTools2.0. Peaks were called from nucleosome-free fragments with MACS2 (-f BAMPE). Consensus peak lists were defined using the reduce function from R/Bioconductor package GenomicRanges (v1.38.0). Peak-based read counts were obtained with the featureCounts function from R/Bioconductor package Rsubread (v2.0.1) and normalised using the calcNormFactors function (method = "TMM") in R/Bioconductor package edgeR (v3.28.1). Differential accessibility analysis was performed with R/Bioconductor package limma (v3.42.2), after applying the voom function.
RNA-seq data were anlaysed using the STAR aligner (v2.7.7a), gene-based read counts were obtained with STAR and normalised by calculating

Transcripts Per Million (TPM). Differential expression analysis was performed using DESeq2 (v1.30.1).
The UCSC LiftOver tool was used to convert mm9 to mm10 coordinates (https://genome.ucsc.edu/cgi-bin/hgLiftOver).
Gene Ontology analysis was performed at http://geneontology.org/.
Gene symbols were obtained for transcripts using Ensembl BioMart (https://www.ensembl.org/biomart/martview).

For manuscripts utilizing custom algorithms or software that are central to the research but not yet described in published literature, software must be made available to editors and reviewers. We strongly encourage code deposition in a community repository (e.g. GitHub). See the Nature Portfolio guidelines for submitting code & software for further information.

Data

Policy information about availability of data

All manuscripts must include a data availability statement. This statement should provide the following information, where applicable:

- Accession codes, unique identifiers, or web links for publicly available datasets
- A description of any restrictions on data availability
- For clinical datasets or third party data, please ensure that the statement adheres to our policy

ATAC-seq data generated in this study have been deposited at Gene Expression Omnibus under accession number GSE195767.
The mass spectrometry proteomics data have been deposited to the ProteomeXchange Consortium via the PRIDE107 partner repository with the dataset identifier PXD039521.
Raw poly-A RNA-sequencing data from WT and Suv39h dn ESCs, corresponding to GSE57092, were obtained from the NCBI Sequence Read Archive (SRA).
UCSC mm10 genome and annotation files were retrieved from Illumina iGenome (http://igenomes.illumina.com.s3-website-us-east-1.amazonaws.com/Mus_musculus/UCSC/mm10/Mus_musculus_UCSC_mm10.tar.gz).
The ENCODE mm10-blacklist.v2 used to filter peaks is available at https://github.com/Boyle-Lab/Blacklist.
Gene Ontology annotations for use in Persues were downloaded from http://annotations.perseus-framework.org (mainAnnot.mus_musculus.txt).
All other relevant data supporting the key findings of this study are available within the article and its supplementary files or from the corresponding author upon reasonable request. Proteomics data are provided in Supplementary Data 1 and 2. Source Data are provided for Figures 1b,c,e-h, 2a-c,e,f, 4b-d, 5a,b (Source Data file) and Supplemental Figures S1e, S2a-d,f,g, S4c-e,g,h, S5b, S6b,c (Supplementary Data 3).

Field-specific reporting

Please select the one below that is the best fit for your research. If you are not sure, read the appropriate sections before making your selection.

☒ Life sciences ☐ Behavioural & social sciences ☐ Ecological, evolutionary & environmental sciences

For a reference copy of the document with all sections, see nature.com/documents/nr-reporting-summary-flat.pdf

Life sciences study design

All studies must disclose on these points even when the disclosure is negative.

Sample size	We used minimum n=3 for the FACS and proteomics since analysis and visualization of the proteomics data with the aid of either volcano plots or a heatmap and hierarchical clustering requires a minimum of three biological replicates for the t-test. The data were highly consistent between replicates such that n=3 was sufficient to define a large number of significant differences.<br>ATAC-seq was performed in duplicate as the minimum required for statistical comparisons whilst keeping sequencing costs down.<br>For experiments involving imaging cells or chromosomes, analysis is from three independent experiments to ensure reproducibility, with the total number of cells or chromosomes specified for each graph.<br>Expression and ChIP qPCR experiments were performed in biological triplicate to ensure reproducibility and allow statistical comparisons.
Data exclusions	There was no exclusion/inclusion of samples in the analysis. All replicate attempts were successful.
Replication	All FACS analysis and imaging experiments were performed in a minimum of three biological replicates for each cell line.<br>Proteomics analysis was performed in three biological replicates, with each analyzed in technical duplicate.<br>Expression and ChIP qPCR measurements were performed in technical triplicate for each of at least three independent biological replicates.<br>Western blots were repeated for three biological replicates.<br>All replicate attempts were successful.
Randomization	Randomization was not relevant to this study as there was no assignment of samples to different experimental groups.
Blinding	Blinding was not relevant since there was no assignment of samples to different experimental groups.

Reporting for specific materials, systems and methods

We require information from authors about some types of materials, experimental systems and methods used in many studies. Here, indicate whether each material, system or method listed is relevant to your study. If you are not sure if a list item applies to your research, read the appropriate section before selecting a response.

Materials & experimental systems

n/a	Involved in the study
☐	☒ Antibodies
☐	☒ Eukaryotic cell lines
☒	☐ Palaeontology and archaeology
☒	☐ Animals and other organisms
☒	☐ Human research participants
☒	☐ Clinical data
☒	☐ Dual use research of concern

Methods

n/a	Involved in the study
☒	☐ ChIP-seq
☐	☒ Flow cytometry
☒	☐ MRI-based neuroimaging

Antibodies

Antibodies used

Primary antibodies: CENP-A (2048S, Cell Signaling, clone C51A7, lot:4), H3K9me3 (07-523, Millipore, lot:2793831) or (07-442, Millipore), H3K27me3 (Ab6002, Abcam, clone mAbcam 6002, lot: 3018864) or (07-449, Millipore), Esrrb (PP-H6705-00, Perseus Proteomics, clone H6705), Sox2 (ab97959, Abcam), H3S10ph (ab5176, Abcam), and Histone H3 (Active Motif 61476, clone 1B1-B2).
Secondary antibodies: anti-mouse-Alexa488 (A11001, Invitrogen), anti-rabbit-Alexa488 (A11008, Invitrogen), anti-mouse-Alexa568 (A11031, Invitrogen), anti-rabbit-Alexa680 (A21109, Invitrogen), anti-mouse-Alexa790 (A11371, Invitrogen).

Validation

CENP-A (2048S):
-Supplier website: (C51A7) Rabbit mAb detects endogenous levels of total mouse CENP-A protein. This antibody does not cross-react with other histone proteins, including Histone H3.
-Validated for IF (Smoak et al, Current Biology, 2016).

H3K9me3 Abs:
-07-523, supplier website: Recognizes Histone H3 containing trimethyl-lysine 9 and, to a lesser extent, dimethyl-lysine 9. MW ~17 kDa. A broad species cross-reactivity is expected.
-07-442, supplier website: Specificity=Trimethyl-histone H3 (Lys9). Validation includes Immunocytochemistry. Broad species cross-reactivity expected, including mouse.
-both validated for IF using Suv39h1/h2 double knockout cells (Djeghloul et al, Stem Cell Reports 2016 and this study).

H3K27me3 Abs:
-Ab6002, supplier website: This antibody is specific for histone H3 tri-methylated at K27. Suitable for ICC/IF. Reacts with mouse.
-07-449, supplier website: Specificity=Trimethylated histone H3 (Lys27) (dot blot tested). Reacts with mouse. Validated in ICC.
-both validated for IF using EED Knockout ESCs (Djeghloul et al, Nature Communications 2020, this study, unpublished data).

Esrrb (PP-H6705-00):
-Supplier specification sheet: This antibody specifically recognizes human ERR beta (ESRRB) and cross reacts with mouse and rat ERR beta. This antibody does not recognize human ERR alpha and gamma. Tested for western blot, immunoprecipitation and immunohistochemistry.
-Validated for ChIP, IF, and WB using Esrrb KO cells (EKOiE ES cells) (Festuccia et al, Nature Cell Biology 2016; Festuccia et al, The Embo Journal 2018, Festuccia et al, Genome Res 2019)

Sox2 (ab97959):
-Supplier website: tested for ICC/IF, reacts with mouse.
-Validated for IF (Percharde et al, Genes Dev, 2012; Djeghloul et al, Nature Communications 2020)

H3S10ph (ab5176):
-Supplier website: Specificity - This antibody is specific for phosho S10 of histone H3. We believe that it does not recognise the non-modified histone - no blocking is seen with the non-phospho peptide. Predicted to work with mouse. Tested for ICC.
-Validated for IF using interphase and metaphase-arrested cells (this study).

H3 (61476):
-Supplier website: validated for WB, wide range of species reactivity predicted.

Eukaryotic cell lines

Policy information about cell lines

Cell line source(s)

Mouse ESCs used in this study: WT ESC (WT26), Suv39h dn (DN57), Suv39h1-EGFP (Suv39h dn ESCs overexpressing full length Suv39h1), from Thomas Jenuwein's Lab (Lehnertz et al, Curr Biol 2003; Velazquez Camacho et al, Elife 2017), and Esrrb-tdTomato ESCs (gift from Nicola Festuccia; Fesrtuccia et al, Nature Cell Biology 2016).
Mouse Embryonic Fibroblasts (MEFs) used in this study were WT (W8), Suv39h dn (D5), WT (clone Eset25 control), Suv39h1/2 -/- (CRISPR clone B1), Setdb1/2 -/- (CRISPR clone A4+OHT) and G9a/Glp -/- (CRISPR clone H7), from Thomas Jenuwein's Lab (Peters et al, Cell 2001; Montavon et al, Nat Commun 2021).

Authentication

All cell lines were tested for Karyotype, genotyped using appropriate primers, and validated by WB and immunofluorescence.

Mycoplasma contamination

All cell lines were tested negative for mycoplasma contamination.

Commonly misidentified lines (See ICLAC register)	No commonly misidentified cell lines were used in this study.

Flow Cytometry

Plots

Confirm that:

☒ The axis labels state the marker and fluorochrome used (e.g. CD4-FITC).

☒ The axis scales are clearly visible. Include numbers along axes only for bottom left plot of group (a 'group' is an analysis of identical markers).

☒ All plots are contour plots with outliers or pseudocolor plots.

☒ A numerical value for number of cells or percentage (with statistics) is provided.

Methodology

Sample preparation	Chromosome sorting:<br>Chromosomes were extracted from the different cell lines and stained with Hoechst 33258 and Chromomycin A3.<br>Chromosomes were examined by flow cytometry using a Becton Dickinson Influx equipped with spatially separated lasers.<br>Hoechst 33258 was excited using a (Spectra Physics Vanguard, air cooled) 355 nm laser with a power output of 350 mW.<br>Hoechst 33258 fluorescence was collected using a 400 nm long pass filter in combination with a 500 nm short pass filter.<br>Chromomycin A3 was excited using a (Coherent Genesis, water cooled) 460 nm laser with a power output of 500 mW.<br>Chromomycin A3 fluorescence was collected using a 500 nm long pass filter in combination with a 600 nm short pass filter.<br>Forward scatter was measured using a (Coherent Sapphire) 488 nm laser with a power output of 200 mW and this was used as the trigger signal for data collection. Chromosomes were sorted at an event rate of 20000 per second. A 70-micron nozzle tip was used along with a drop drive frequency set to ~96 KHz and the sheath pressure was set to 65 PSI.<br><br>PI staining:<br>Cells were fixed with ice-cold 70% ethanol, washed twice with PBS and resuspended in staining buffer containing 0.05 mg/ml of PI, 1 mg/ml RNaseA, and 0.05% NP40. Samples were incubated for 10 min at room temperature (RT) and 20 min on ice.
Instrument	Chromosomes: Becton Dickinson Influx equipped with spatially separated air cooled lasers.<br>PI staining: BD Fortesa flow cytometer.
Software	BD FACS software (v1.2.0.142, Influx); BD DIVA (v8.0.1, Fortesa).
Cell population abundance	Gating strategy and percentages of chromosome 19 and X is provided in the manuscript.<br>Purity of individual chromosome sort was assessed by DNA FISH with mouse chromosome 19- or X-specific paints. 99-100% sample purity was achieved.<br>Gating strategy and percentages of each phase of cell cycle are provided in the manuscript.
Gating strategy	Chromosomes were first gated on a plot of high Hoechst 33258 vs low Forward scatter signal to gate out debris and clumps. This first gate was then used to create a chromosome karyotype by plotting Hoechst 33258 vs Chromomycin A3 fluorescence. Gating strategies for chromosome sorting and cell cycle analysis are provided in the manuscript.

☒ Tick this box to confirm that a figure exemplifying the gating strategy is provided in the Supplementary Information.
